# Penicillin-Susceptible, Oxidase-Negative, Nonhemolytic, Nonmotile* Bacillus megaterium* in Disguise of* Bacillus anthracis*

**DOI:** 10.1155/2017/2578082

**Published:** 2017-02-26

**Authors:** Shih Keng Loong, Boon Teong Teoh, Jefree Johari, Chee Sieng Khor, Juraina Abd-Jamil, Siti Sarah Nor'e, Nur Izyan Samsudin, Noor Syahida Azizan, Che Norainon Yaacob, Asma Anati CheMatSeri, Nur Hidayana Mahfodz, Sazaly AbuBakar

**Affiliations:** ^1^Tropical Infectious Diseases Research & Education Centre (TIDREC), Faculty of Medicine, University of Malaya, 50603 Kuala Lumpur, Malaysia; ^2^Department of Medical Microbiology, Faculty of Medicine, University of Malaya, 50603 Kuala Lumpur, Malaysia

## Abstract

*Bacillus anthracis* is a bacterial pathogen of major concern. The spores of this bacteria can survive harsh environmental conditions for extended periods and are well recognized as a potential bioterror weapon with significant implications. Accurate and timely identification of this* Bacillus* species in the diagnostic laboratory is essential for disease and public health management. Biosafety Level 3 measures and ciprofloxacin treatment were instituted when* B. anthracis* was suspected from a patient with gangrenous foot. 16S rDNA sequencing was performed to accurately identify the suspected bacterium, due to the superiority of this method to accurately identify clinically isolated bacteria.* B. megaterium* was identified as the causative agent and the organism was subsequently treated as a Biosafety Level 2 pathogen.

## 1. Introduction

The genus* Bacillus* comprises organisms that are Gram-positive and rod-shaped and form endospores [1]. Two species,* B. anthracis* and* B. cereus*, are clinically significant pathogens, causing anthrax and nausea and diarrhea, respectively [2]. Anthrax is classically characterized into three forms of disease, cutaneous, gastrointestinal, and inhalational anthrax, each with different fatality rates [3, 4]. Recently, a new infection route, described as injectional anthrax, has been associated with nonspecific symptoms and high fatality rates in sporadic outbreaks among drug users in Europe [4]. Cutaneous anthrax usually results from contact with infected animals and it is the most common infection route [3, 4]. Gastrointestinal anthrax is rare and is usually acquired through consumption of contaminated food [3, 4]. Inhalational anthrax is recognized as the most severe form of the disease and it has to be treated urgently for any chance of survival, with mortality rates approaching 100% if untreated [3, 4]. Anthrax is recognized as having national security importance because it has a relatively short incubation period, causes rapid progression of disease, and can also be utilized as a bioterror weapon [3]. There are, however, limitations in species differentiation within the genus* Bacillus* as some species share similar biochemical and antigenic profiles [5]. The anthrax attack on the United States of America in 2001 accentuated the threat of* B. anthracis* [5] and the need for accurate* Bacillus* species identification in the clinical setting. In the present study, we report the isolation of a* Bacillus* species, which was suspected to be* B. anthracis* but was subsequently confirmed to be* B. megaterium*.

## 2. Case Report

A 47-year-old diabetic woman was admitted to the University Malaya Medical Centre in May 2007 with gangrene on the right foot. She experienced fever and her right toe was amputated. Following that, tissue specimens were extracted from the amputated toe. Blood drawn on admission was sent to the laboratory and the findings showed high levels of triglyceride (2.2 mmol/L; normal range: 0.45 to 1.7 mmol/L) and alkaline phosphatase (178 IU/L; normal range: 50 to 136 IU/L). A complete blood count revealed an elevated white blood cell count of 11.9 × 10^9^/L (normal range: 4.0 to 11.0 × 10^9^/L). Blood urea nitrogen and anion gap levels were 72 mmol/L (normal range: 2.5 to 6.4 mmol/L) and 25 mmol/L (normal range: 10 to 20 mmol/L), indicating a kidney problem. Levels of C-reactive protein were high at 5.1 mg/dL (normal range: 0 to 0.8 mg/dL).

Tissue specimens from the amputated toe were incubated on blood and MacConkey agar in aerobic and anaerobic chambers at 37°C for 24 h. A mixed culture was observed after 24 h in blood agar incubated under aerobic and anaerobic conditions, with one colony type producing green pigmentation surrounding the colonies and the other producing pale yellow colonies. Both isolates were individually subcultured onto blood agar followed by incubation in the same conditions for 24 h. The pale yellow colonies were unable to grow on MacConkey agar and showed scant growth anaerobically. The hemolytic isolate producing green pigmentation was again cultured in blood agar. It was suspected to be* Pseudomonas aeruginosa* and was subsequently confirmed by growth in asparagine broth and 16S rDNA sequencing using previously described methods [6].

**Figure 1:**
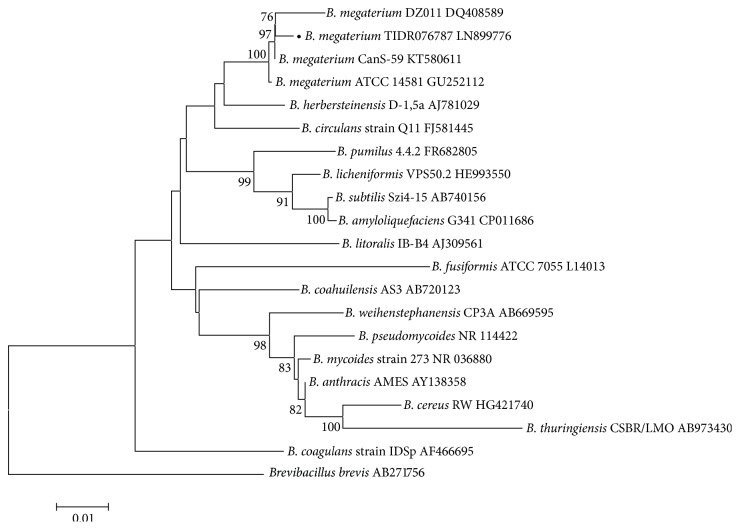
Neighbor-joining phylogenetic tree of representative* Bacillus* spp. The* B. megaterium* isolate obtained in the present study is indicated by a solid black circle before its name. Accession numbers are indicated after the respective* Bacillus* spp. names.* Brevibacillus brevis* was used as an outgroup. Numbers at nodes indicate bootstrap values (%) for 1,000 replicates. The scale bar indicates nucleotide substitutions per site.

The pale yellow isolates were oxidase-negative, nonhemolytic, nonmotile, and spore-forming Gram-positive rods. These biochemical tests were repeated once to confirm the results. String test was negative, further confirming the Gram-positive organism. Based on the phenotypic characteristics of the organism resembling* B. anthracis*, hospital authorities were informed and the still febrile patient was given intravenous ciprofloxacin intervention on day 5 after admission. The isolate was immediately treated as a risk group 3 pathogen and all further sample processing was performed in a Biosafety Level (BSL) 3 containment facility. Disc diffusion tests revealed that the suspected* B. anthracis* isolate was susceptible to amikacin, cefoperazone, ceftazidime, ciprofloxacin, erythromycin, gentamicin, imipenem, netilmicin, penicillin, piperacillin, and vancomycin. Subsequent species identification of this isolate was performed using 16S rDNA sequencing [6]. Consensus sequences of the partial 16S rDNA gene (accession number LN899776) explicitly identified the* Bacillus* isolate as* B. megaterium*, as shown in Figure 1. Intravenous ciprofloxacin was discontinued after 9 days of treatment and the patient subsequently recovered and was discharged. All subsequent manipulation of the isolate was performed in a BSL 2 containment facility.

## 3. Discussion

Two different bacteria species,* P. aeruginosa* and* B. megaterium*, were isolated from the patient's foot tissues, in agreement with a previous study that found that diabetic foot infections usually yield more than one isolate [9]. That same study also found that* P. aeruginosa* is a commonly isolated bacterium from diabetic wounds, with 13% prevalence, and that patients with chronic wounds are likely to be colonized by* P. aeruginosa* [9]. Even though the patient's laboratory findings corroborated the clinical presentation of a diabetic patient, elevated levels of white blood cells and C-reactive protein in serum samples also implied the presence of a systemic infection [10].

Initial suspicion of* B. anthracis* was based on the isolate's nonhemolytic and nonmotile growth properties. The isolate was also oxidase-negative and penicillin-susceptible, further strengthening the suspicion. Unlike in the United States, where after* Bacillus* colonies are identified as nonmotile and nonhemolytic the isolate is treated as highly suspicious for* B. anthracis* and is sent to the Laboratory Response Network for Bioterrorism for confirmation [2], there is no such requirement in Malaysia. In lieu of that, the hospital authorities were immediately informed and biosafety control measures commensurable with a risk group 3 pathogen were initiated. 16S rDNA sequencing was selected over other anthrax identification methods such as gamma phage susceptibility [5], detection of specific cell wall and capsular antigens [5], and real-time PCR [11] because of the limitation of expertise to perform these tests and the superiority of 16S rDNA sequencing to identify rare clinical bacteria isolates [6].

Although the patient did not show classical anthrax symptoms and had no contact with animals, the possibility of anthrax was considered based on a recent case of septicemic anthrax in an intravenous drug user that had no indication of classical symptoms [12] and whose symptoms were similar to the patient in this study. The possibility of anthrax was also considered based on* B. anthracis* being easily acquired as a soil contaminant [3]. While the recovery of* B. anthracis* in any infection is deemed a serious health threat, the finding of* B. megaterium* in clinical specimens should not be brushed aside as an insignificant contaminant [1]. We found four previously reported clinical cases related to* B. megaterium* [1, 2, 7, 8] from the literature, listed in chronological order in Table 1. Including the isolate in this study, three out of the five cases were wound infections. Of the five patients, four were female and one was male, with a mean age of 37 years (range: 23–50 years). In our investigation,* B. megaterium* was also isolated from the patient's wound.* B. megaterium* was also implicated in a case of ovarian mass torsion [2], which suggested that the bacterium entered the bloodstream and then localized to the ovary. A similar hypothesis was also proposed in a case of* B. megaterium*-related brain abscess [7], highlighting the pathogenic potential of this organism. This hypothesis is also supported by the demonstration of* B. megaterium* attachment and subsequent invasion of human erythrocytes [13]. Also,* B. megaterium* isolated from honey was found to have toxin-encoding genes such as enterotoxin-T, the HBL complex, and the NHE complex, often associated with foodborne illnesses [13], accentuating the virulence capability of this* Bacillus* species.

The patient in our investigation was treated empirically with ciprofloxacin since it was considered the most suitable antibiotic for the treatment of anthrax [3]. It was also deemed safe to discontinue the treatment upon the identification of* B. megaterium* [3], which was also sensitive to ciprofloxacin. Patients in the other* B. megaterium*-associated cases [1, 2, 8] were also successfully treated with ciprofloxacin. Acquisition of the bacteria in our patient was likely through contact with contaminated soils or roadside plants that have been shown to harbor bacteria including* B. megaterium*,* B. anthracis*, and* B. cereus* [14]. Since the organism was isolated from foot tissues, it was likely that the patient contracted the bacteria through microabrasions in the skin [1]. Taken together,* B. megaterium* clearly possesses pathogenic potential in humans, although its clinical outcome is relatively favorable and it is still susceptible to common antibiotics. However,* B. megaterium* cannot be absolutely ascertained as the cause of infection in this case as* P. aeruginosa* was also concurrently isolated.

## 4. Conclusion

Although* B. megaterium* cannot be definitely proven to be the cause of infection in this case study, this bacterium evidently displayed capabilities to cause disease based on reports in the literature. Thus, the finding of this bacterial species in other clinical specimens should not be ignored and warrants further investigation. Brushing aside* B. megaterium* as an insignificant contaminant may result in serious ramifications for affected patients.

## Figures and Tables

**Table 1:** Summary of *B. megaterium*-associated clinical cases in the literature.

Year of report	Patient data	Clinical diagnosis	Treatment and clinical outcome	Reference
Current study	47-year-old woman with diabetes	Gangrenous toe	Treated with ciprofloxacin; clinically stable; discharged with no sign of disease	This study

2015	50-year-old woman with psoriasis and a 3-month history of fever and headache	Brain abscesses	Clinically stable after penicillin was used	Guo et al. [7]

2011	25-year-old immunocompetent woman with no fever and chills	Primary cutaneous infection	Treated with ciprofloxacin; clinically stable; discharged with no sign of disease	Duncan and Smith [1]

2006	23-year-old man	Lamellar keratitis	Treated with amikacin, azithromycin, ciprofloxacin, and clarithromycin; clinically stable; patient had 20/15 visual acuity on the affected eye 1 year after surgery	Ramos-Esteban et al. [8]

2003	40-year-old immunocompetent woman with no chills	Ovarian mass torsion	Treated with clindamycin, ciprofloxacin, and rifampin; clinically stable; discharged with no sign of disease	Dib et al. [2]
